# Structural Analysis and Insights into the Oligomeric State of an Arginine-Dependent Transcriptional Regulator from *Bacillus halodurans*

**DOI:** 10.1371/journal.pone.0155396

**Published:** 2016-05-12

**Authors:** Young Woo Park, Jina Kang, Hyun Ku Yeo, Jae Young Lee

**Affiliations:** Department of Life Science, Dongguk University-Seoul, Ilsandong-gu, Goyang-si, Gyeonggi-do, Republic of Korea; Centro Nacional de Biotecnologia - CSIC / CIF Q2818002D, SPAIN

## Abstract

The arginine repressor (ArgR) is an arginine-dependent transcription factor that regulates the expression of genes encoding proteins involved in the arginine biosynthesis and catabolic pathways. ArgR is a functional homolog of the arginine-dependent repressor/activator AhrC from *Bacillus subtilis*, and belongs to the ArgR/AhrC family of transcriptional regulators. In this research, we determined the structure of the ArgR (Bh2777) from *Bacillus halodurans* at 2.41 Å resolution by X-ray crystallography. The ArgR from *B*. *halodurans* appeared to be a trimer in a size exclusion column and in the crystal structure. However, it formed a hexamer in the presence of L-arginine in multi-angle light scattering (MALS) studies, indicating the oligomerization state was dependent on the presence of L-arginine. The trimeric structure showed that the C-terminal domains form the core, which was made by inter-subunit interactions mainly through hydrophobic contacts, while the N-terminal domains containing a winged helix-turn-helix DNA binding motif were arranged around the periphery. The arrangement of trimeric structure in the *B*. *halodurans* ArgR was different from those of other ArgR homologs previously reported. We finally showed that the *B*. *halodurans* ArgR has an arginine-dependent DNA binding property by an electrophoretic mobility shift assay.

## Introduction

The arginine metabolism pathway is essential for various organisms and strictly controlled by the arginine repressor (ArgR) in bacteria. ArgR does not only regulate the transcription of nearby genes of the arginine biosynthesis regulon in the presence of L-arginine [1], but is also involved in activation of the arginine catabolic pathways including arginase pathway [2], arginine deiminase pathway [3], and arginine succinyltransferase pathway [4]. ArgR binds to the well-conserved DNA sequence called the ARG box in the promoter regions of the genes involved in the arginine biosynthesis and catabolic pathways in the presence of high concentration of L-arginine [5].

The crystal structures of ArgR homologs from bacterial species, including *Escherichia coli* (*Ec*ArgR), *Bacillus subtilis* (*Bsu*AhrC), *Bacillus stearothermophilus* (*Bst*ArgR), *Mycobacterium tuberculosis* (*Mtb*ArgR) and *Vibrio vulnificus* (*Vu*ArgR), have been determined [6–9]. The structures of ArgR in a complex with the DNA operator have been determined from *M*. *tuberculosis* and *B*. *stearothermophilus* [9–11]. The structural data of full-length ArgR from *B*. *stearothermophilus* has provided the first view of an intact ArgR protein and has contributed to understanding the differences observed in the quaternary organization of each subunit between apo and arginine-bound forms [8, 12]. The ArgR monomer consists of highly conserved two domains separated by a protease accessible linker; the N-terminal domain is classified by a winged helix-turn-helix DNA binding domain and the C-terminal domain is responsible for oligomerization and arginine binding [13, 14]. Each ArgR monomer assembles into either trimers or hexamers, being controlled by the protein concentration and the presence of the L-arginine corepressor. There are 6 L-arginines located in the trimer-trimer interface of the ArgR hexamer and enable the two trimers to dimerize each other, acting as molecular glues [6, 12]. The two adjacent N-terminal domains of a hexameric form interact with the one ARG box. The ARG box is a pair of slightly imperfect palindrome sequences. The consensus sequence was described as TNTGAATWWWWATTCANW in *E*. *coli*, CATGAATAAAAATKCAAK in *B*. *subtilis* and AWTGCATRWWYATGCAWT in *Streptomycetes* (where W = A or T, K = G or T, R = A or G, Y = T or C, M = A or C, N = any base) [15–17].

In case of *E*. *coli*, ArgR mainly exists in a hexameric state regardless of the presence or absence of L-arginine [18]. Although *Bsu*AhrC and *Bst*ArgR repressors are purified mainly in a trimeric state [8, 19, 20], they can be assembled into a hexameric state, which is favoured at a high protein concentration and in the presence of L- arginine [21].

The bacterial ArgR protein can be categorized into three major classes based on the arginine dependence and the ARG box sequence specificity [22]. Class I ArgR proteins from *E*. *coli*, *Salmonella typhimurium*, and *Marsupella profunda* bind to the target operator containing the ARG box in a highly arginine-dependent manner and have narrow target sequence specificity. The Class I ArgR proteins mainly exist in a hexameric state in spite of low protein concentration and the absence of L-arginine [4, 5, 18, 23]. The ArgR proteins from Gram-positive *Bacillus* and *Streptomyces* species belong to Class II. The Class II ArgR proteins exist in equilibrium between trimeric and hexameric states and promote transition from trimer to hexamer when protein concentration is high and/or L-arginine is present. They have a broad target sequence specificity and their binding to DNA is dependent on L-arginine moderately [7, 8, 19, 24]. The ArgR proteins from *Thermotoga neapolitana* and *Thermotoga maritima* belong to Class III, which can interact with cognate operators containing the ARG box sequence as well as heterologous ARG box. The Class III ArgR shows poor target specificity and its DNA binding is marginally influenced by L-arginine [22].

**Fig 1:**
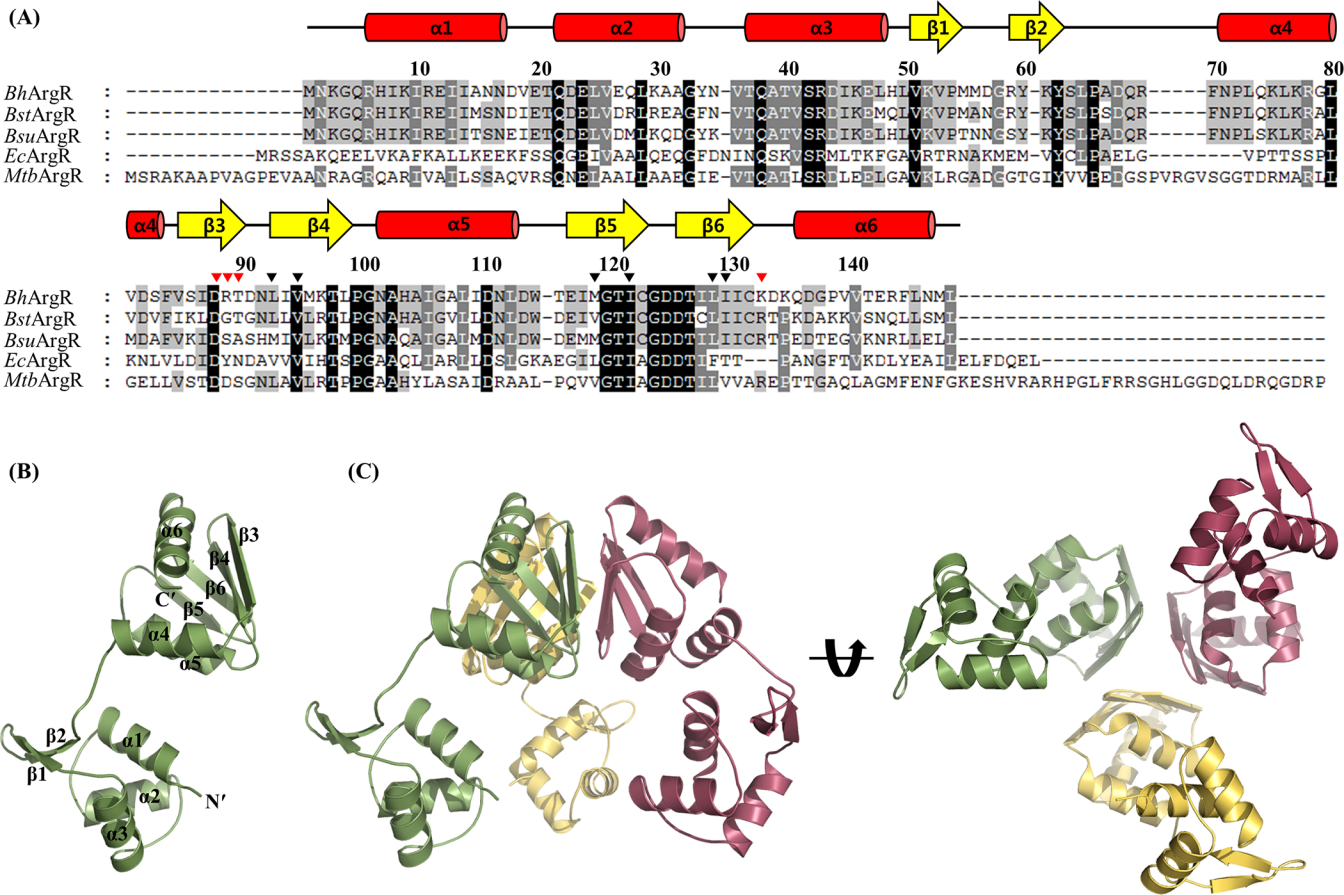
The overall structure of *Bh*ArgR. (A) A multiple sequence alignment of *Bh*ArgR and represented homologous ArgR proteins from *B*. *stearothermophilus*, *B*. *subtilis*, *E*. *coli*, and *M*. *tuberculosis*. Every 10th residue is shown above the sequence of *Bh*ArgR. Highly conserved residues and partially conserved residues are shaded in black and grey, respectively. The residues involved in trimeric core with hydrophobic interactions and hydrogen bonds are indicated as black and red closed triangles, respectively. (B) The overall structure of *Bh*ArgR monomer. (C) The trimeric structure of *Bh*ArgR generated by a crystallographic three-fold symmetry through C-terminal domain (Each subunit is coloured in green, red, and yellow).

The ArgR homologue (BH2777) from *Bacillus halodurans* (*Bh*ArgR) has been identified and it encodes a protein of 149 amino acid residues with 72% sequence identity to *Bsu*AhrC. Further sequence comparison of *Bh*ArgR with other ArgR proteins from *B*. *stearothermophilus*, *E*. *coli*, and *M*. *tuberculosis* showed 73%, 28%, and 33% sequence identity, respectively (Fig 1A). Although the crystal structures of ArgR from bacterial species have been determined, the biological roles of oligomeric states of ArgR and binding ability to the DNA operator with the L-arginine corepressor are not entirely understood. To provide a structural basis for a better understanding of the oligomeric state, DNA recognition, and L-arginine dependency, we hereby report the crystal structure of the apo form of ArgR from *B*. *halodurans* (*Bh*ArgR). The structure reveals that the *Bh*ArgR exists in a trimeric form showing quite a different domain arrangement from the trimeric forms of other bacterial species. Furthermore, our electrophoretic mobility shift assay shows that *Bh*ArgR is capable of binding to cognate DNA containing ARG box sequence in the presence of L-arginine.

## Materials and Methods

### Structure determination and refinement

DNA cloning, expression, purification, crystallization, and data collection of *Bh*ArgR have been described previously. The crystals belonged to the space group I23, with unit cell parameters a = b = c = 104.68 Å, containing one molecule of ArgR monomer in the asymmetric unit [25]. The initial model was obtained by molecular replacement with the program *PHASER* [26], as a starting model using a *Bst*ArgR structure (PDB code 1B4A) with 73% sequence identity [8]. The molecular replacement was not successful using the whole chain of the model (residues 4–149) as a starting model. When the model was split into two domains, two separate models of *Bh*ArgR consisting of the N-terminal domain (residues 4–65) and the C-terminal domain (residues 68–149) were successfully identified in the asymmetric unit.

A further model building was manually conducted using the *COOT* program [27] and refined with the *PHENIX* program suite [28]. The refined model of *Bh*ArgR, which accounts for 146 residues in monomer, one molecule of *1*,*2*-propanediol, and 74 water molecules, gave R_work_ and R_free_ values of 18.2% and 23.8%, respectively, for data in the resolution range of 28.0–2.41 Å (Table 1). A random set of 5% of the reflections was excluded from the refinement for cross-validation of the refinement strategy. The quality of the model was checked using *MolProbity* [29]. All residues were allowed in the favoured region of the Ramachandran plot. The refinement statistics are presented in Table 1. The coordinates and structure factors have been deposited in the Protein Data Bank under accession number 5CJ9 for the *Bh*ArgR structure.

**Table 1:** Structural solution and Refinement.

Space group	I23
Unit cell parameters	
a, b, c (Å)	104.68, 104.68, 104.68
α, β, γ (°)	90.00, 90.00, 90.00
Resolution range (Å)	28.0–2.41
Completeness (%)	99.97
No. of reflections, working set	7,193
No. of reflections, test set	349
Final *R*_cryst_ ^a^	18.2
Final *R*_free_	23.8
No. of non-H atoms	
Protein	1150
*1*,*2*-propanediol	5
Water	74
Total	1229
R.m.s. deviations	
Bonds (Å)	0.009
Angles (°)	1.240
Average *B* factors (Å^2^)	
Protein	52.5
Ligand	47.9
Water	57.6
Ramachandran plot ^b^	
Most favored (%)	96.5
Allowed (%)	3.5

^a^ *R*_cryst_ = Σ | |*F*_obs_|–|*F*_calc_| | / Σ |*F*_obs_|, where *R*_free_ was calculated from a randomly chosen 10% of reflections, which were not used for structure refinement, and *R*_cryst_ was calculated for the remaining reflections.

^b^ Determined using *Molprobity*.

### Electrophoretic mobility shift assay

To assess the DNA binding ability of the purified *Bh*ArgR, we predicted DNA operator sequences by using the *PreDetector* program [30] based on the ArgR/AhrC recognition signal candidate sequences [31]. A 119-bp cognate DNA containing its own promoter region was prepared by polymerase chain reaction (PCR) using the primers Bh119F (5′-CCCAGAATACGCTAAGACAAC-3′) and Bh119R (5′-TTTATACAGGCCTTTTTTTATGC-3′). A 250-bp noncognate DNA was isolated by PCR using *Thermoplasma acidophilum* genomic DNA as a template. The reaction buffer was composed of 50 mM Tris-HCl (pH 7.5), 200 mM NaCl, 10 mM MgCl_2_, 10 mM CaCl_2_, and 10% glycerol (*v/v*). The *Bh*ArgR proteins were added to the reaction mixture prior to the DNA. All reaction mixtures were incubated on ice for 40 min. Then, the reaction mixtures were resolved on a 6% pre-chilled non-denaturing polyacrylamide gel in Tris-borate-EDTA (TBE) buffer (pH 8.8) with or without the addition of 10 mM L-arginine at 100 V for 40 min. After electrophoresis at 4°C, the gel was visualized using an EMSA staining kit (Life Technology).

### Size-exclusion chromatography with multi-angle light scattering (SEC-MALS)

SEC-MALS experiments were performed using a fast protein liquid chromatography system (GE Healthcare) connected to a Wyatt MiniDAWN TREOS MALS instrument and a Wyatt Optilab rEX differential refractometer. A Superdex-200 10/300 GL (GE Healthcare) gel filtration column pre-equilibrated with 20 mM Tris-HCl (pH 8.0), 200 mM NaCl, 2 mM MgCl_2_, and 1 mM DTT was normalized using ovalbumin (43 kDa) as a protein standard. The *Bh*ArgR protein was injected (3~6 mg ml^-1^, 0.2 ml) at a flow rate of 0.5 ml min^-1^ in the presence or absence of 10 mM L-arginine. The data were evaluated using the Zimm model for static light scattering data fitting and represented using an EASI graph with a UV peak in the *ASTRA V* software (Wyatt).

## Results and Discussion

### The overall structure of *Bh*ArgR monomer

The *Bh*ArgR protein was overexpressed, purified, and crystallized in the absence of L-arginine corepressor. The crystal structure of *Bh*ArgR was determined by molecular replacement at 2.41 Å resolution. The structure was refined to crystallographic R_work_ and R_free_ values of 18.2% and 23.8%, respectively, with good geometry. The final model (PDB code 5CJ9) contained 146 amino acid residues of the monomer, one molecule of *1*,*2*-propanediol, and 74 water molecules in the asymmetric unit, and the model was validated using *MolProbity* [29]. The three N-terminal residues (Met1, Asp2 and Lys3) were disordered in the crystal structure and were not visible on the electron density map. The structure of *Bh*ArgR monomer was elongated with approximate dimensions of 40 Å × 30 Å × 60 Å (Fig 1B). The *Bh*ArgR monomer formed a dumbbell shape consisting of two distinct domains, N and C-terminal domains connected by a protease accessible linker. The N-terminal domain (residues 4–65) contained three α-helices and a pair of antiparallel β-strands. The helices α2 and α3 together with their intervening loop formed a winged helix-turn-helix DNA binding motif that belongs to a large family of transcription factors. The C-terminal domain (residues 71–149) included α/β fold with four antiparallel β-strands (β3, β4, β5 and β6) flanked by three α-helices (α4, α5 and α6) on one side.

The *Bh*ArgR structure formed a symmetric trimer through hydrophobic interactions facing with the other side of four antiparallel β-strands in each subunit (Fig 1C). Intramolecular interaction such as hydrogen bonding, salt bridge, and hydrophobic contact did not exist between the N and C-terminal domains. The five residues (Val51, Val53, Arg59, Pro65 and Phe70) in the N-terminal domain and linker region form a small hydrophobic core which is similar to the *Bst*ArgR structure [8]. The linker region (residues 66–70) between two domains had a poor density and high temperature factors (average B factor 63.4). Therefore, the N-terminal DNA binding domains were highly mobile while the C-terminal domains were assembled into an oligomeric state.

### The trimeric structure of the *Bh*ArgR

**Fig 2:**
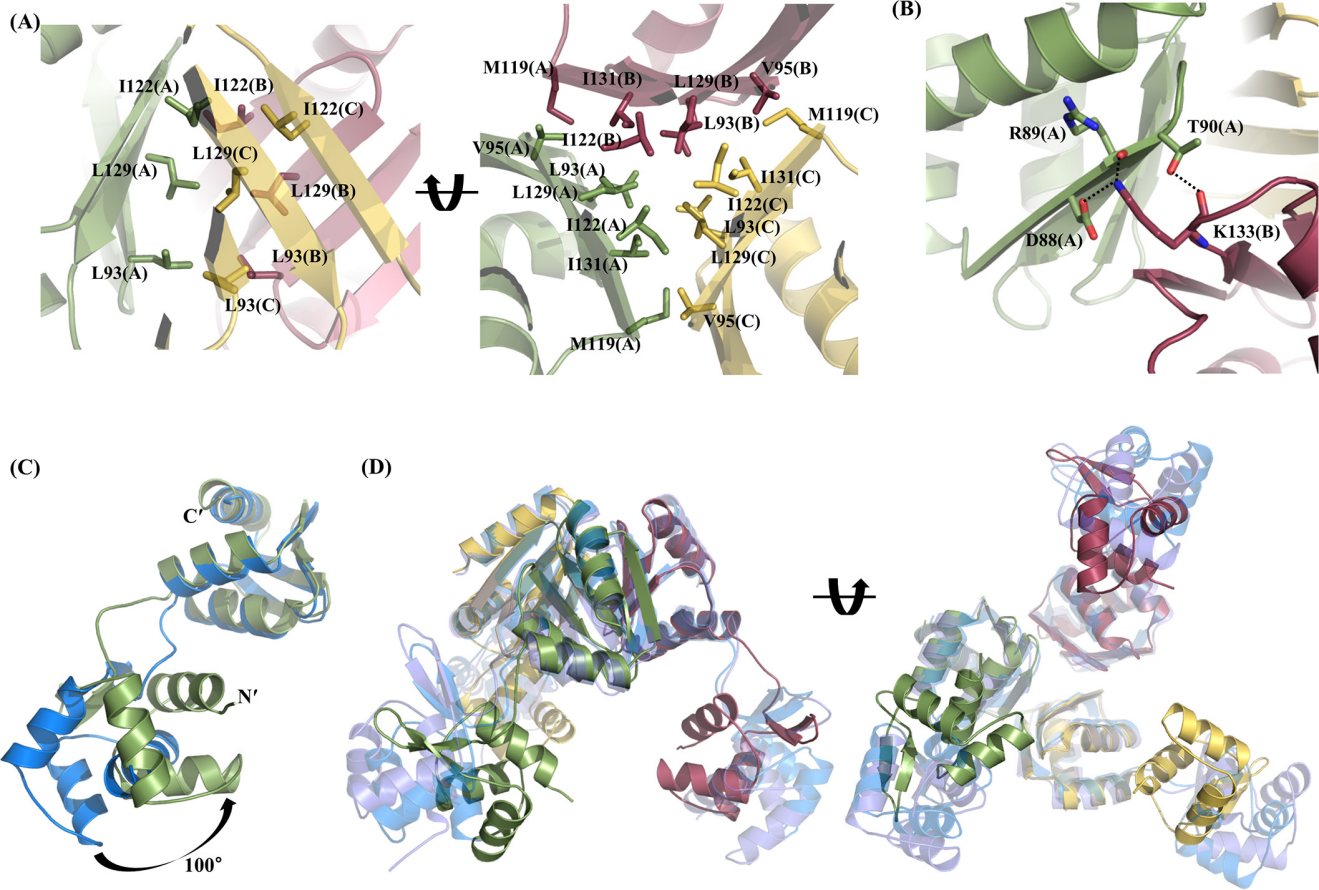
The oligomeric structure of the *Bh*ArgR and structural comparison with other homologous proteins. (A) The interface of the *Bh*ArgR trimer makes a hydrophobic core by the highly conserved residues Leu93, Leu129, and Ile122 of each subunit along the three-fold axis (left panel). The hydrophobic core is reinforced by the adjacent residues Val95, M119, and Ile131 of each subunit (right panel). (B) The hydrogen bond interactions are shown around the hydrophobic core in the *Bh*ArgR trimer. (C) Comparison with the N-terminal domains based on superposition of C-terminal domains of *Bh*ArgR (in green) and *Bst*ArgR (in blue). (D) Comparison with the trimeric structure of *Bh*ArgR and other homologous proteins based on superposition of C-terminal domains (*Bst*ArgR in blue; *Bsu*AhrC in violet).

Although a monomer of *Bh*ArgR existed in each asymmetric unit of the crystal, it could be generated into a trimeric structure, which was a tripod-like shape with approximate dimensions of 80Å × 65 Å × 60 Å. The trimeric structure of *Bh*ArgR was composed of three monomers related by a crystallographic three-fold symmetry through the antiparallel β-sheets of their C-terminal domains (Fig 1C). The N-terminal domains in trimeric structure made crystal contacts with other N-terminal domains in a symmetry-related trimer. The solvent accessible surface area buried at the interface in this trimeric structure was about 1,400 Å^2^ (~ 5.6% of the trimeric surface area), and 22 amino acid residues in each monomer were involved in this interface (*PDBePISA* protein-protein interaction server; http://www.ebi.ac.uk/msd-srv/prot_int/). The trimeric interface was mainly made of the hydrophobic residues in C-terminal domain. In particular, the trimeric core was composed of three hydrophobic residues, Leu93, Leu129, and Ile122 of each subunit. Each residue was involved in a hydrophobic contact with the same residues in the other subunits along three-fold axis, which made three layers of hydrophobic interactions (Fig 2A). The trimeric hydrophobic core was reinforced by the adjacent hydrophobic residues, Ile131, Val95, and Met119. The trimeric interface was also contributed by several hydrogen bonds (Asp88(A)Oδ1–Lys133(B)Nζ, Arg89(A)O–Lys133(B)Nζ, Thr90(A)Oγ1–Lys133(B)O, Lys133(A)O–Thr90(C)Oγ1, Lys133(A)Nζ–Arg89(C)O, Lys133(A)Nζ–Asp88(C)Oδ1, Asp88(B)Oδ1–Lys133(C)Nζ, Arg89(B)O–Lys133(C)Nζ, and Thr90(B)Oγ1–Lys133(C)O) (Fig 2B). These results indicated that *Bh*ArgR existed as a trimeric form in the absence of L-arginine.

**Fig 3:**
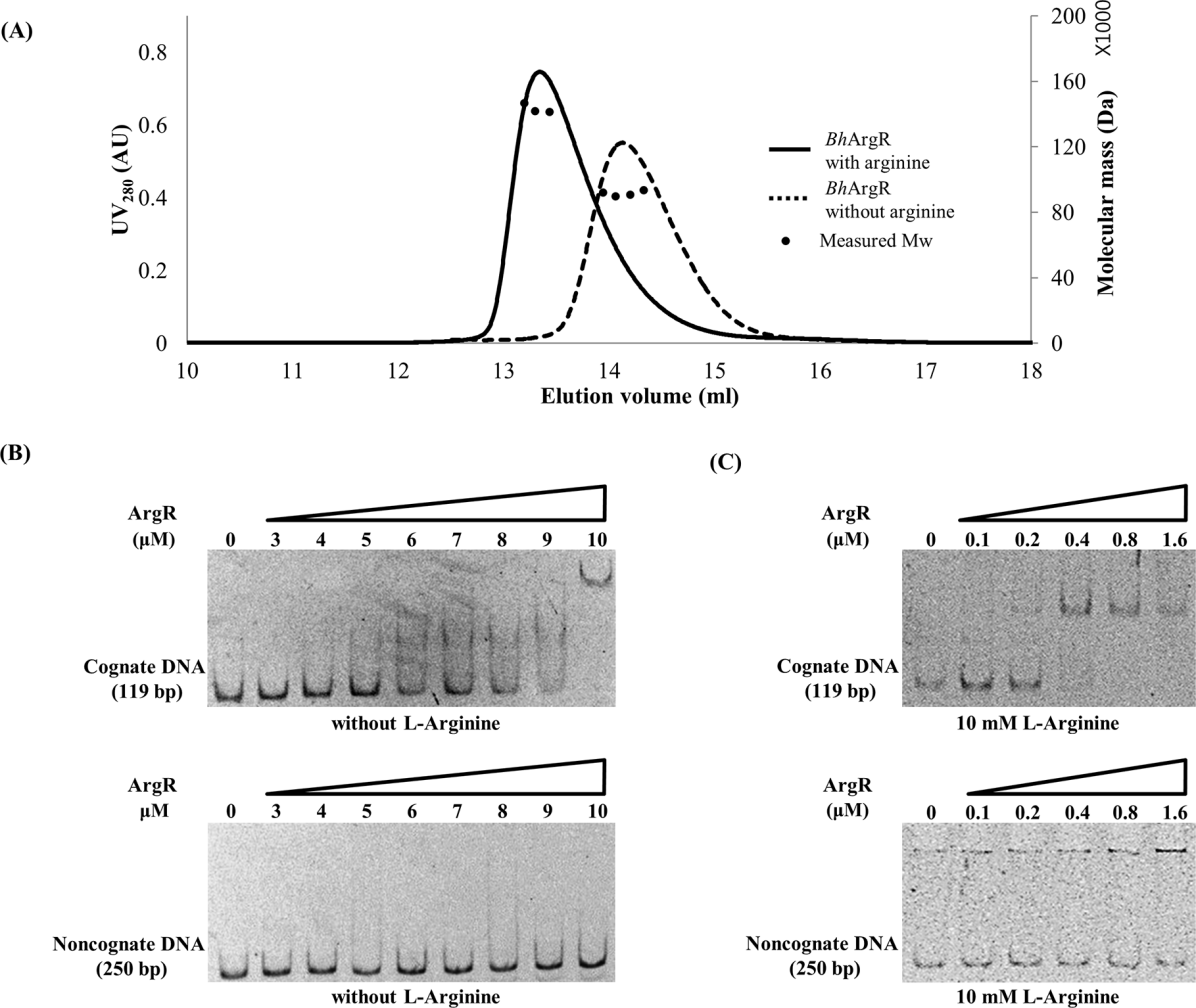
The oligomeric state and DNA binding activity of *Bh*ArgR. (A) SEC-MALS profiles of *Bh*ArgR in the presence (solid line)/absence (dashed line) of L-arginine (10 mM). Small circles represent the calculated molecular mass (Da) in right ordinate axis. The normalized UV absorbance at 280 nm is drawn with the solid and dashed lines in left ordinate axis. (B) EMSA was performed using cognate 119-bp DNA containing one ARG box positioned at -36 upstream from *argG* gene without L-arginine (upper). 250-bp noncognate DNA was used as a negative control (bottom). All lanes contained 20 nM cognate or noncognate dsDNA; lane 1, no protein; lanes 2–9, 3, 4, 5, 6, 7, 8, 9, and 10 μM *Bh*ArgR protein. (C) EMSA was performed using same DNA of (B) with L-arginine. All lanes contained 20 nM cognate (upper) or noncognate (bottom) dsDNA, and 10 mM L-arginine; lane 1, no protein; lanes 2–6, 0.1, 0.2, 0.4, 0.8, 1.6 μM *Bh*ArgR protein.

In order to understand how L-arginine affects the oligomeric state between trimer and hexamer, we performed the size-exclusion chromatography with multi-angle light scattering (SEC-MALS) to determine the absolute molecular weights of *Bh*ArgR in solution. In the SEC-MALS experiment, the *Bh*ArgR protein samples were fractionated on a Superdex 200 column and monitored. The molecular masses of fractions in the elution could be estimated relative to the protein standard (ovalbumin). In the absence of L-arginine, the retention volume of *Bh*ArgR was 14.1 ml, corresponding to 90.8±1.0 kDa. In the presence of 10 mM L-arginine, the *Bh*ArgR was eluted earlier, 13.3 ml, corresponding to 154±0.6 kDa (Fig 3A). The hexa-histidine tagged *Bh*ArgR had a molecular mass of about 114 kDa as a hexamer and 57 kDa as a trimer. The SEC-MALS results showed that the earlier peaks with L-arginine represented the hexameric form while the later eluted peaks without L-arginine represented the trimeric form of *Bh*ArgR. The larger experimental molecular masses of the *Bh*ArgR oligomer could be explained due to their tripod-like assembly of the N-terminal domains. These data suggested that the *Bh*ArgR mainly exist as a trimer in the absence of L-arginine and promote the transition to a hexamer in the presence of L-arginine in solution.

### Structural comparison with other proteins

**Fig 4:**
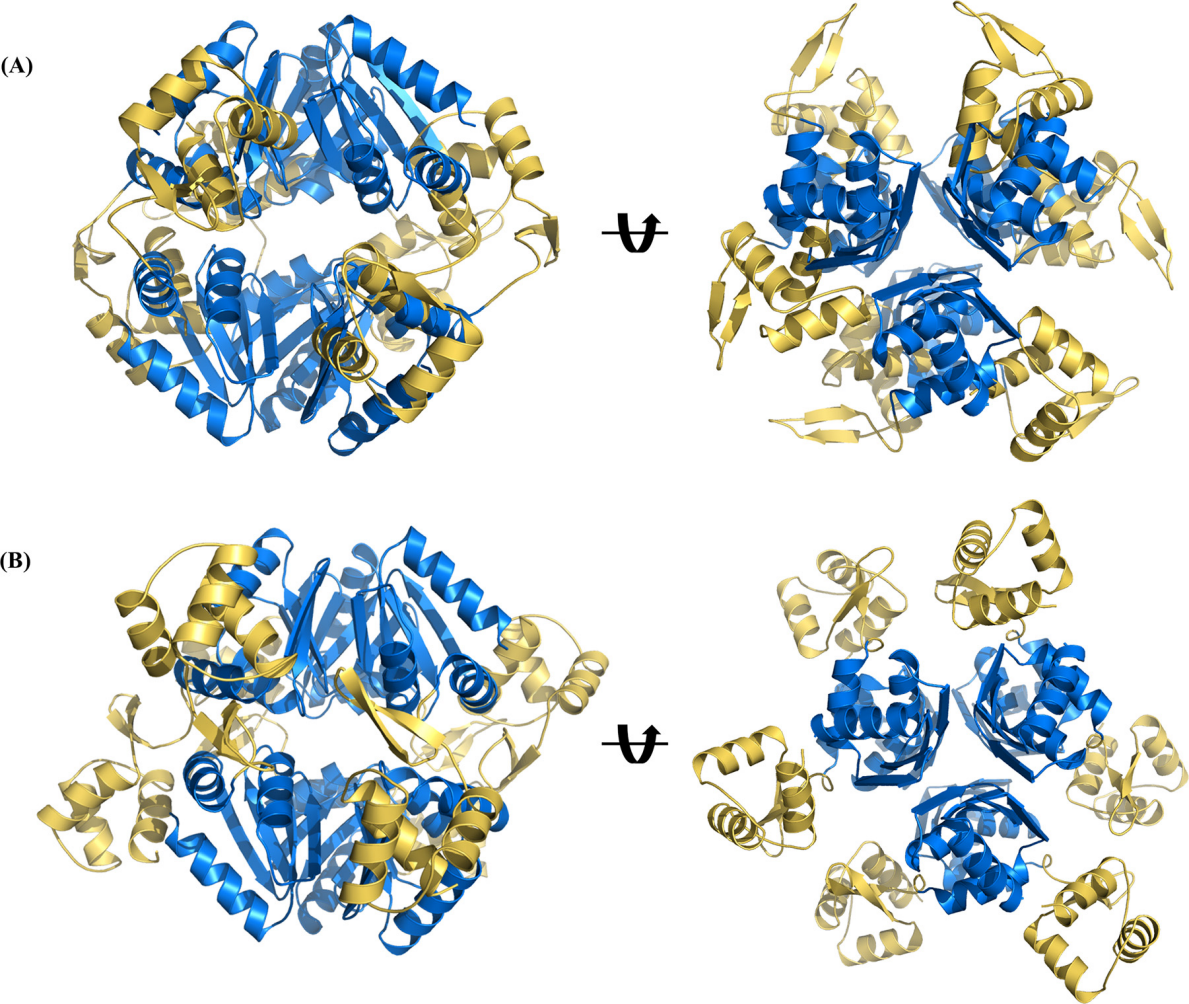
Comparison with the hexameric structures of *Bh*ArgR and *Bst*ArgR. (A) The hypothetical hexameric structure of *Bh*ArgR was generated based on the *Bst*ArgR hexamer. The *Bh*ArgR cannot assemble into a hexamer due to the clashes between the N-terminal domains and C-terminal domains. (N-terminal domains in yellow; C-terminal domains in blue) (B) The hexameric structure of *Bst*ArgR (PDB code, 1B4A).

Although the other previously determined full-length ArgR/AhrC structures have been reported as a hexameric state, forming an interlocked dimer of trimers, the trimeric structure of *Bh*ArgR could be generated by three-fold crystallographic symmetry, whereas the hexameric structure could not be generated. When the hexameric structure was generated, the arrangements of the N-terminal domains of one trimer clashed with the C-terminal domains of the other trimer and interrupted assembling trimers into a hexamer (Fig 4).

We compared the sequence and structural similarities of the arginine repressors from various organisms using the *Clustal Omega* [32] and *DALI* server [33]. The three full-length ArgR homologues from *B*. *subtilis*, *B*. *searothermophilus*, and *M*. *tuberculosis* were best matched with the *Bh*ArgR; (i) *Bst*ArgR [11] (PDB entry 1B4A; r.m.s. deviation of 1.0 Å for 80 equivalent Cα in residues 4–149, and Z-score of 17.2), (ii) *Bsu*AhrC [8] (PDB entry 1F9N; r.m.s. deviation of 4.7 Å for 102 equivalent Cα in residues 4–149, and Z-score of 17.2), and (iii) *Mtb*ArgR [10] (PDB entry 3LAP; r.m.s. deviation of 6.0 Å for 114 equivalent Cα in residues 4–149, and Z-score of 15.4).

We further elaborated the structural similarity search with individual domains of *Bh*ArgR. Using the N-terminal domain (residues 4–65) alone, the result was similar that obtained using the whole structure of *Bh*ArgR. The highest structural similarity was obtained with the *Bst*ArgR [11] (PDB entry 1B4A; r.m.s. deviation of 0.7 Å for 62 equivalent Cα in residues 4–65, and Z-score of 13.8). The second highest similarity was found with the *Bsu*AhrC [8] (PDB entry 1F9N; r.m.s. deviation of 0.5 Å for 62 equivalent Cα in residues 4–65, and Z-score of 12.9). Using C-terminal domain (residues 71–149) alone, the highest Z-scores were obtained with the *Bst*ArgR [11] (PDB entry 1B4A; r.m.s. deviation of 0.8 Å for 79 equivalent Cα in residues 71–149, and Z-score of 17.3), and with the *Bsu*AhrC [8] (PDB entry 1F9N; r.m.s. deviation of 0.8 Å for 79 equivalent Cα in residues 71–149, and Z-score of 17.3).

Although the sequence and structure of each domain were well conserved among the organisms, the relative position of the each domain was considerably different (Figs 1A, 2C and 2D). The distinct conformation arose from the flexible linker (residue 66–70) and the absence of the corepressors, L-arginine. When the C-terminal domains of *Bh*ArgR were superimposed with those of other ArgR structures, each N-terminal domain was headed toward the trimeric core by about 100° rotation centred at linker region (Fig 2C and 2D) and interrupted the assembly of trimers into a hexameric form. These results suggested that the N-terminal domains of the *Bh*ArgR trimer should be rearranged in order to be a hexameric conformation and the corepressor, L-arginine, may enhance the *Bh*ArgR to be a hexameric state.

### DNA binding ability of the *Bh*ArgR

To assess whether *Bh*ArgR binds to DNA, we performed an EMSA using 119-bp DNA containing one ARG box sequence with or without L-arginine. The ARG box is positioned -36 upstream from *argG* gene encoding argininosuccinate lyase involved in arginine transport and metabolism. We found that the cognate DNA was shifted at relatively high concentration of *Bh*ArgR in the absence of L-arginine (Fig 3B). However, the DNA binding ability of *Bh*ArgR was enhanced in the presence of 10 mM L-arginine (Fig 3C). We did not observe a significant DNA complex formation with noncognate DNA either with or without L-arginine (Fig 3B and 3C). This result demonstrated that *Bh*ArgR binds to its own ARG box DNA sequence in an L-arginine-dependent manner *in vitro*.

## Conclusion

We determined the structure of *B*. *halodurans* ArgR in apo form. Structural analyses revealed that *Bh*ArgR could exist in trimeric form, which had an unusual domain arrangement compared with hexameric form of other ArgR structures. The SEC-MALS experiment indicated that *Bh*ArgR mainly existed in a trimeric state in solution in the absence of L-arginine. However, the corepressor L-arginine enhanced the *Bh*ArgR to assemble into a hexameric state. EMSA results demonstrated that *Bh*ArgR was capable of binding to cognate DNA containing ARG box sequence in the presence of L-arginine *in vitro*. Structural analyses and biochemical data suggested that *Bh*ArgR and its closely related homologues could assemble into a hexamer and bind the cognate DNA in an L-arginine-dependent manner. Taken together, the *B*. *halodurans* ArgR may function as an arginine-dependent transcriptional regulator.

## Supporting Information

S1 File *B*. *halodurans* ArgR coordinate. (PDB)

S2 File *B*. *halodurans* ArgR structure factor. (MTZ)

S3 File Validation report of *B*. *halodurans* ArgR structure. (PDF)
